# The wavelet power spectrum of perfusion weighted MRI correlates with tumor vascularity in biopsy-proven glioblastoma samples

**DOI:** 10.1371/journal.pone.0228030

**Published:** 2020-01-23

**Authors:** Lukas T. Rotkopf, Benedikt Wiestler, Christine Preibisch, Friederike Liesche-Starnecker, Thomas Pyka, Dominik Nörenberg, Stefanie Bette, Jens Gempt, Kolja M. Thierfelder, Claus Zimmer, Thomas Huber

**Affiliations:** 1 Department of Diagnostic and Interventional Neuroradiology, Klinikum rechts der Isar, Technical University Munich, Munich, Germany; 2 Department of Neuropathology, Institute of Pathology, Technical University Munich, Munich, Germany; 3 Institute of Clinical Radiology and Nuclear Medicine, Medical Faculty Mannheim, University of Heidelberg, Mannheim, Germany; 4 Department of Diagnostic and Interventional Radiology and Neuroradiology, Universitaetsklinikum Augsburg, Augsburg, Germany; 5 Department of Neurosurgery, Klinikum rechts der Isar, Technical University Munich, Munich, Germany; 6 Institute of Diagnostic and Interventional Radiology, Pediatric Radiology and Neuroradiology, University Medical Center Rostock, Rostock, Germany; Henry Ford Health System, UNITED STATES

## Abstract

**Background:**

Wavelet transformed reconstructions of dynamic susceptibility contrast (DSC) MR perfusion (wavelet-MRP) are a new and elegant way of visualizing vascularization. Wavelet-MRP maps yield a clear depiction of hypervascular tumor regions, as recently shown.

**Objective:**

The aim of this study was to elucidate a possible connection of the wavelet-MRP power spectrum in glioblastoma (GBM) with local vascularity and cell proliferation.

**Methods:**

For this IRB-approved study 12 patients (63.0+/-14.9y; 7m) with histologically confirmed IDH-wildtype GBM were included. Target regions for biopsies were prospectively marked on tumor regions as seen on preoperative 3T MRI. During subsequent neurosurgical tumor resection 43 targeted biopsies were taken from these target regions, of which all 27 matching samples were analyzed. All specimens were immunohistochemically analyzed for endothelial cell marker CD31 and proliferation marker Ki67 and correlated to the wavelet-MRP power spectrum as derived from DSC perfusion weighted imaging.

**Results:**

There was a strong correlation between wavelet-MRP power spectrum (median = 4.41) and conventional relative cerebral blood volume (median = 5.97 ml/100g) in Spearman's rank-order correlation (κ = .83, p < .05). In a logistic regression model, the wavelet-MRP power spectrum showed a significant correlation to CD31 dichotomized to no or present staining (p = .04), while rCBV did not show a significant correlation to CD31 (p = .30). No significant association between Ki67 and rCBV or wavelet-MRP was found (p = .62 and p = .70, respectively).

**Conclusion:**

The wavelet-MRP power spectrum derived from existing DSC-MRI data might be a promising new surrogate for tumor vascularity in GBM.

## Introduction

Perfusion-weighted imaging (PWI) is frequently used in neuro-oncologic magnetic resonance imaging (MRI) and allows further glioma characterization. Established PWI parameters like cerebral blood volume (CBV) have been shown to correlate with tumor vascularity, glioma grade and survival [1,2]. Moreover, CBV might be a promising biomarker in assessing response to anti-angiogenic agents like Bevacizumab, a FDA-approved drug for treatment of recurrent glioblastoma (GBM) [3,4]. CBV is reflected by the area under the bolus curve for each voxel time course and is therefore a measure of the general amount of blood in a specific brain volume. In contrast, wavelet transformation of the time course (wavelet-MRP) allows the extraction of vessel-specific spectra from perfusion data. This method was first demonstrated in computed tomography perfusion (CTP) and was recently adapted to dynamic susceptibility contrast (DSC) PWI [5,6]. Wavelet-PWI yields reproducible color-coded maps with a clear depiction of vessel-specific tumor regions in GBM and a strong suppression of background structures leading to an excellent image contrast [7]. The maximum of the wavelet power spectrum (WPS) is a sensitive parameter incorporating both perfusion magnitude and bolus shape, leading to marked signal amplification in vessel-rich tissue. Therefore, the wavelet-MRP is a complementary parameter that reflects a different aspect of PWI than the standard parameters cerebral blood flow, mean transit time or time to peak. Wavelet-MRP is not redundant to these standard PWI parameters and can possibly add additional clinical benefits in the initial evaluation and the follow-up of patients with GBM. Even though the hypothesis that wavelet-MRP truly correlates with tumor vascularity seems natural from a biophysical point of view, further histological evidence is needed. However, validation of this hypothesis is challenging and complex since a detailed analysis ideally requires region-specific tissue samples. The aim of this study was to investigate if the wavelet-MRP is linked to endothelial and proliferation markers in a small sample of GBM patients that obtained DSC MRI and underwent targeted biopsies prior to neurosurgical tumor resection.

## Materials and methods

### Patient population

For this IRB approved study, a total of 12 patients (63.0 ± 14.9y; 7m) with histologically confirmed IDH-wildtype GBM were included from February 2013 to July 2016. All subjects gave their informed written consent to targeted biopsies during neurosurgical GBM resection as part of an earlier study at our maximum care university hospital site. Data from this cohort got previously published in a different study [8]. The study was approved by the ethics commission of the Technical University of Munich and conducted in accordance with the ethical standards of the 1964 Declaration of Helsinki and its later amendments.

### MR imaging

All patients were examined on a 3 Tesla Biograph mMR scanner (Siemens Medical Solutions, Germany). The acquisition protocol included a high-resolution T2-weighted fluid-attenuated-inversion-recovery (FLAIR) sequence, a T1-weighted magnetization prepared rapid gradient echo (MPRAGE) sequence (spatial resolution 1 × 1 × 1 mm; TR/TE 9/4 ms; inversion time TI 900 ms) and single‐shot gradient-echo echo planar imaging (EPI) (TR/TE/α = 1500 ms, / 30 ms,/ 90°, voxel size 1.8 × 1.8 × 4 mm^3^, 20 slices, 80 dynamics; bolus injection of 15 ml Gd‐DTPA after a prebolus of 7.5 ml) for dynamic acquisitions of DSC MRI. No pre-bolus was applied.

### Co-registration strategies

In order to allow volume of interest (VOI) based analysis, co-registration of all processed perfusion maps was performed on the T1w MPRAGE as target using MATLAB R2017a (The MathWorks, Massachusetts, USA) and SPM12 (https://www.fil.ion.ucl.ac.uk/spm/software/spm12/, accessed on August 6, 2019). Visual inspection of all maps revealed no significant spatial deviations.

### Perfusion analysis

As described previously, conventional perfusion analysis was performed by a validated custom MATLAB program [9]. In addition, segmentation of anatomical maps into gray matter (GM) and white matter (WM) using standard SPM12 algorithms was used. Using an AUC-based leakage-correction method, relative cerebral blood volume (rCBV) maps were derived from raw DSC MRI perfusion data [9,10].

### Wavelet analysis

Using a custom Python 3.5 script, a continuous wavelet transform with the *Paul* wavelet was applied to the bolus time course corresponding to each voxel of the motion-corrected perfusion dataset [5,7]. The maximum power coefficient was then obtained by calculating the maximum of the squared coefficient matrix over all scales. Briefly, following [7], with $I(\vec{r},t)$ being the measured intensity signal dependent on both voxel location $\vec{r}$ and time t, the wavelet transform is calculated by

$$W[I\;(\vec{r},t)](u,s)=\int_{0}^{\infty }I(\vec{r},t)\;\psi_{0}^{*}\;(\frac{t-u}{s})\;\mathrm{dt}$$

where $\psi_{0}^{*}$ stands for the mother wavelet, which must fulfill several conditions, namely by being quadratintegrable, having zero first moment and a value of zero at the origin. Here, the so-called *Paul wavelet* of moment m = 1 was used[7,11].

$$\psi_{0}(p)=\frac{{\left(2i\right)}^{m}m!}{\sqrt{\pi \;(2\pi )!}}{\left(1-ip\right)}^{-(n+1)}$$

**Fig 1:**
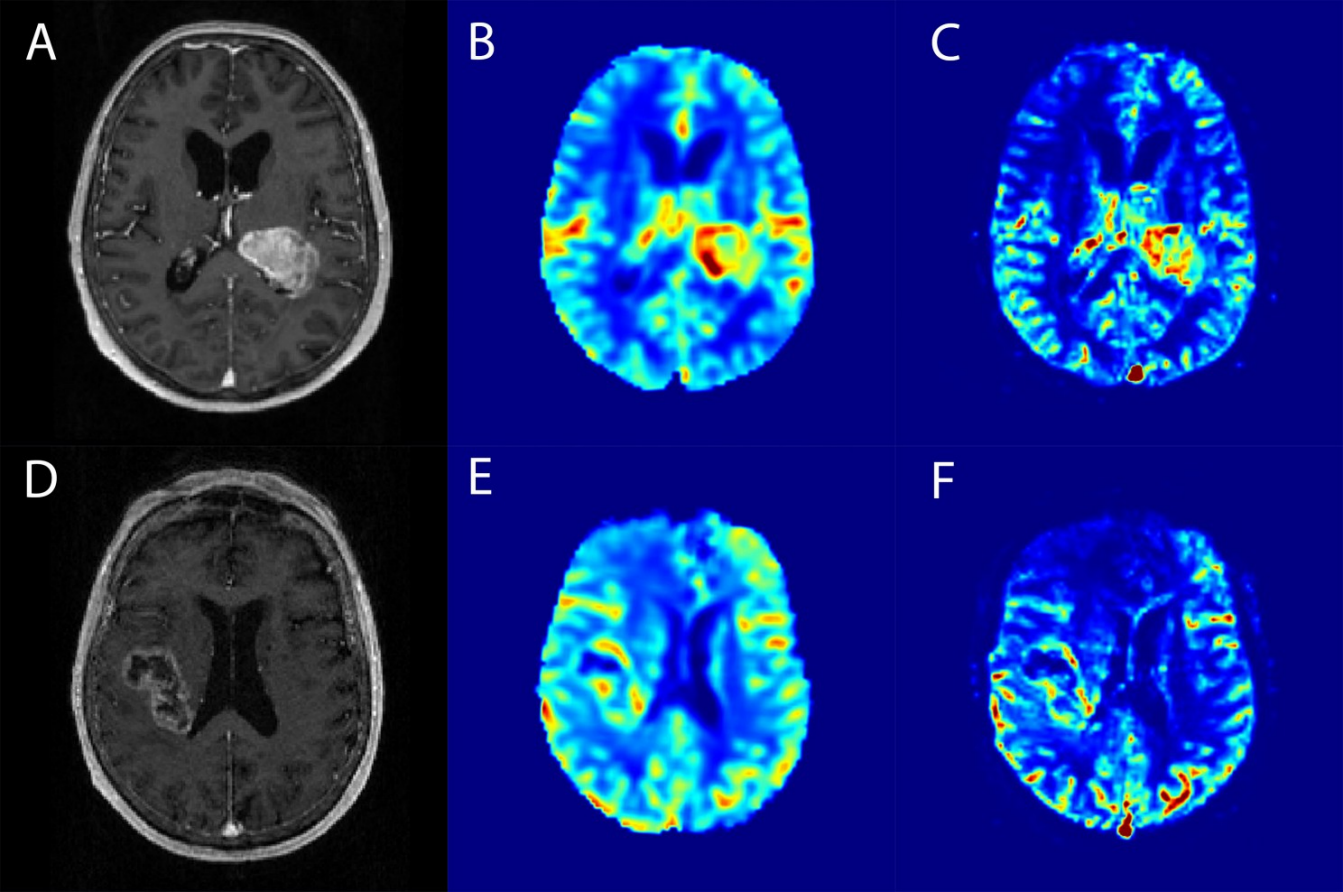
Example images of on spatially corresponding axial processed MR data: contrast-enhanced T1w anatomical images (A, D), relative CBV (B, E), and wavelet-MRP (C, F).

This yields the *wavelet power spectrum* (WPS) for each voxel, namely a matrix of size S x T [5]. Maximal and minimal scale parameters were automatically determined to allow optimal sampling[12]. Finally, the maximum of the WPS in both time and order was interpreted as the perfusion magnitude parameter wavelet-MRP (Fig 1) [5].

$$\mathrm{wavelet}‐\mathrm{MRP}=\underset{u.s}{\max}\left({\left|W[I\;(\vec{r},t)](u,s)\right|}^{2}\right)$$

To allow better inter-method comparison, the signal for each patient was then normalized to the basal mean wavelet power coefficient over the segmented white matter, effectively rescaling all values.

### Targeted biopsies

**Fig 2:**
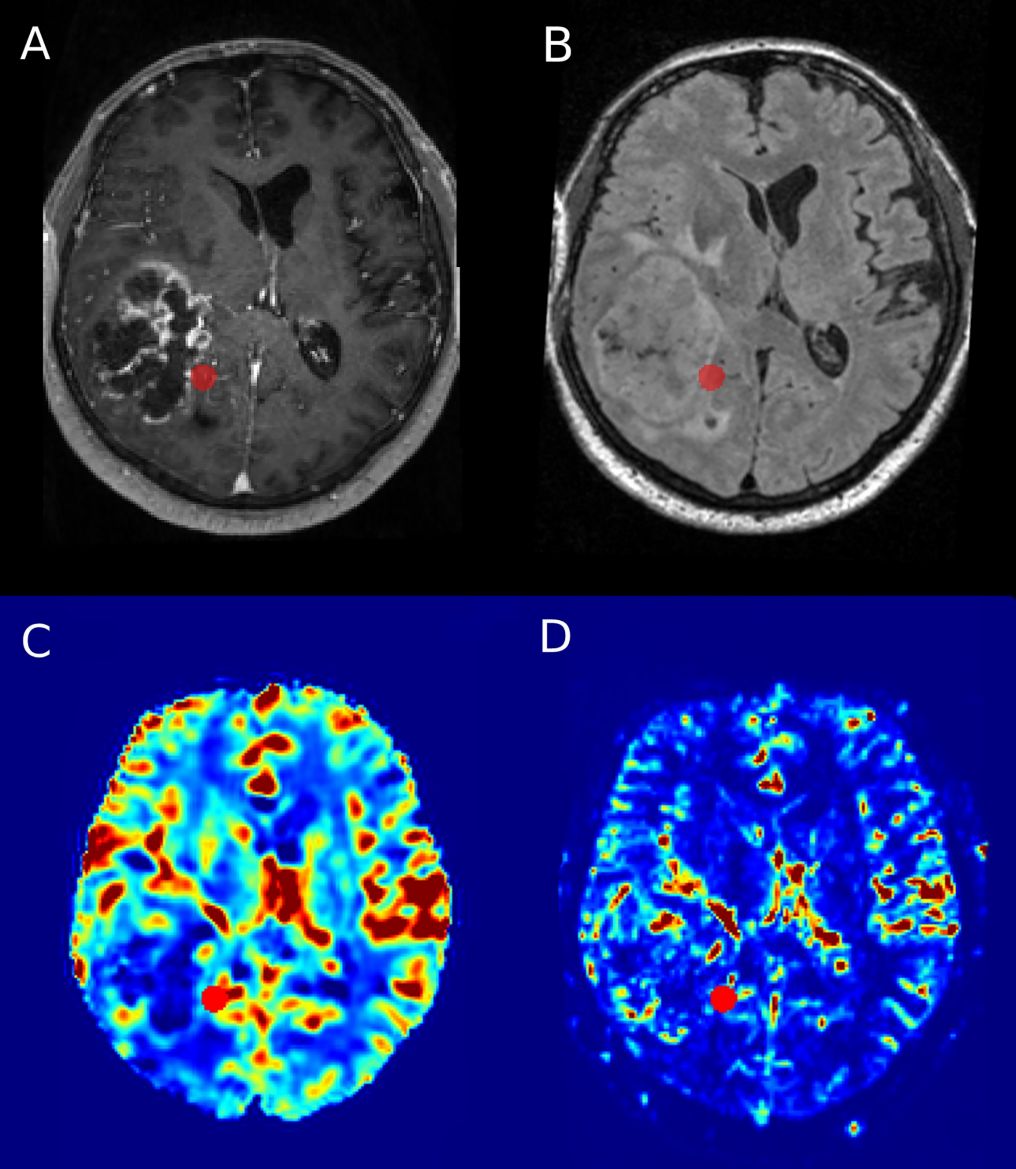
Illustration of VOI placement on spatially corresponding axial contrast-enhanced T1w image (A), FLAIR (B), relative CBV (C) and wavelet-MRP (D).

As described previously [8], VOIs were defined prospectively in consensus with the operating neurosurgeon on the T1w MPRAGE and T2w FLAIR sequences (Fig 2). Each biopsy location was marked with a round VOI of 5 mm diameter. Intraoperatively, cylindrical tissue samples with a diameter between 1 and 4 mm were obtained from these locations with the aid of an image-guided stereotactic biopsy system (VarioGuide, BrainLab). The tissue samples were immediately placed in 4% buffered saline solution and embedded in paraffin within 2–6 hours.

### Immunohistochemistry

**Fig 3:**
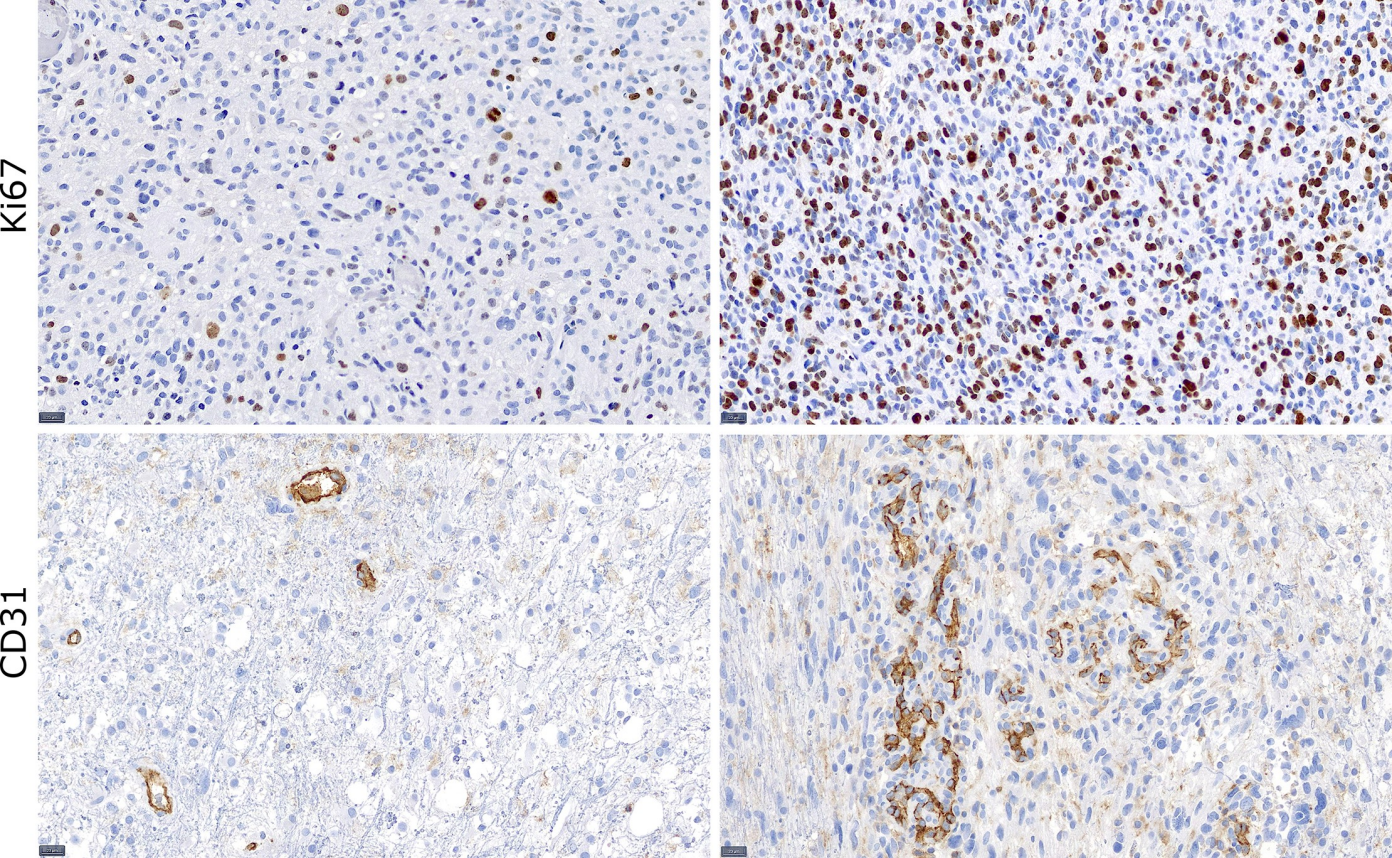
Exemplary histologic specimens of targeted tumor biopsies in a 20-fold magnification (black bars equal 20 μm). The upper row shows Ki67 immunohistochemistry to determine the proliferative activity with a low percentage of 8% proliferating cells (left) and high percentage of 40% proliferating cells (right). The lower row shows CD31 immunohistochemistry to determine the amount of endothelial cells with a low vessel density (score 1, left) and a high vessel density (score 3, right).

Immunohistochemistry was performed using a fully automated staining system (Ventana BenchMark ULTRA; Ventana Medical Systems; Tucson; USA) as described previously [8]. After heat-induced epitope unmasking in pH 8.4 buffer at 95°C for 32 minutes, the samples were incubated with peroxidase and afterwards charged with anti-Ki67 (monoclonal, mouse; Clone JC70A, dilution 1:50; DakoCytomation Denmark A7S, Denmark) or anti-CD31 (polyclonal, rabbit, dilution 1:100; Thermo Fischer Scientific, USA) antibodies, respectively. 3,3’-diaminobenzidine- (DAB-) based OptiView DAB IHC Detection Kit (Ventana Medical Systems) was used for antibody detection subsequently. At last, counterstaining with Meyer’s Hämalaun was performed. Positive controls were used for quality insurance. Due to the inherent difficulties in obtaining tissue samples corresponding to predetermined spatial locations during brain surgery [13], gross appearance and presence of malign tissue as noted by the neuropathologist was compared to the expected composition at the prospective location. Only samples which showed no significant difference in expected and obtained composition were included in the final analysis. Therefore, all samples with histopathologically visible malign cells which were taken from tumor-free regions as seen on imaging and vice versa were excluded. Hereby, 16 samples were excluded due to significant mismatch. The stainings were evaluated by two neuropathologists. The number of CD31-positive vascular proliferates staining was judged on a 4-point scale (0 = no vascular proliferates, 1 = few, 2 = several, 3 = many vascular proliferates). Ki67 staining was assessed by obtaining the ratio of Ki67 expressing cells and all visible cells per microscope field. Fig 3 shows examples for CD31 and Ki67 scoring. Ki67 was additionally analyzed in the diagnostic samples of the resection specimen as estimated mean value. Additionally, the maximum proliferation index per sample was analyzed.

### Volume-of-interest-based correlations

VOI-based comparisons between calculated perfusion values and immunohistochemical staining was done both with rCBV and wavelet-MRP, and Ki67 and CD31 staining, respectively. The mean signal intensity was calculated for each included VOI. These values were subsequently compared to the histological parameters. In addition, perfusion indices were averaged over the visible tumor region. In order to do so, the gross tumor volume (GTV) including necrosis zones was manually segmented on contrast-enhanced T1w maps and the mean values of rCBV and wavelet-MRP over all included pixels calculated. The relationship between rCBV and wavelet-MRP was quantified using Spearman’s rank-order correlation coefficient.

### Statistics and software

All illustrations were created with GIMP 2.8.14 (www.gimp.org) and ITK-SNAP 3.6.0, which was also used for segmentation. Statistical analyses were done with IBM SPSS 23.0 (IBM, Armonk, NY, USA) and R (R Foundation for Statistical Computing, Vienna, Austria). Mixed logistic and linear regression analysis was used to test association strength between variables. For all models except those including GTV, patient identity was entered as random effect. AIC and marginal R^2^ were used as indices of goodness of fit. As addition of the random effect had no effect on tested significances, ROC curves were calculated from fixed effect models only. All metric or nonnormally distributed variables are reported as median. P values less than .05 were considered significant and are marked with an asterisk.

## Results

### Patient and tissue sample characteristics

The initial patient population consisted of 12 patients (7 men) with a mean age of 63.0 ± 14.9 years, all with histopathologically confirmed IDH-wildtype glioblastoma. One to four biopsies were taken from each patient, resulting in a total of 43 biopsies. After exclusion of 16 (37%) non-matching samples, 27 (63%) remained for the final analysis. From these samples, 18 (67%) were taken from tumor tissue, 6 (22%) from surrounding edema and 3 (11%) from microscopically healthy brain tissue. Detailed patient and sample characteristics can be found in Table 1.

**Table 1:** Baseline characteristics patient and biopsy characteristics. Values are presented as count (percentage) for categorial and median (interquantile range) for ordinal or continuous variables.

	Overall (N = 13)		
**Patient Data**	** **	** **	
Age	63.0	(14.9)	
Female sex	5	(41.7%)	
**Biopsy Data**			
Samples per patient	1–4		
All samples	43		
Matching samples	27	(62.8%)	
Location			
CET	18	(66.7%)	
Edema	6	(22.2%)	
Tumor-free tissue	3	(11.1%)	
**Histopathological Data**			
CD31			
Absent staining	8	(29.6%)	
Positive staining	19	(70.4%)	
MIB-1			
Absent staining	3	(11.1%)	
Proliferation Index	14%	(14%)	
**Perfusion Data**			
wavelet-MRP			
CET	5.58	(2.74–7.61)	
Edema	1.36	(0.85–5.15)	
Tumor-free tissue	3.82	(3.56–4.12)	
rCBV			
CET	6.69	(4.76–9.03)	
Edema	3.76	(1.65–5.52)	
Tumor-free tissue	5.04	(4.44–6.05)	

CET contrast enhancing tissue, wavelet-MRP wavelet-transformed Magnetic Resonance Perfusion, rCBV relative Cerebral Blood Volume

### Association between rCBV and wavelet-MRP

**Fig 4:**
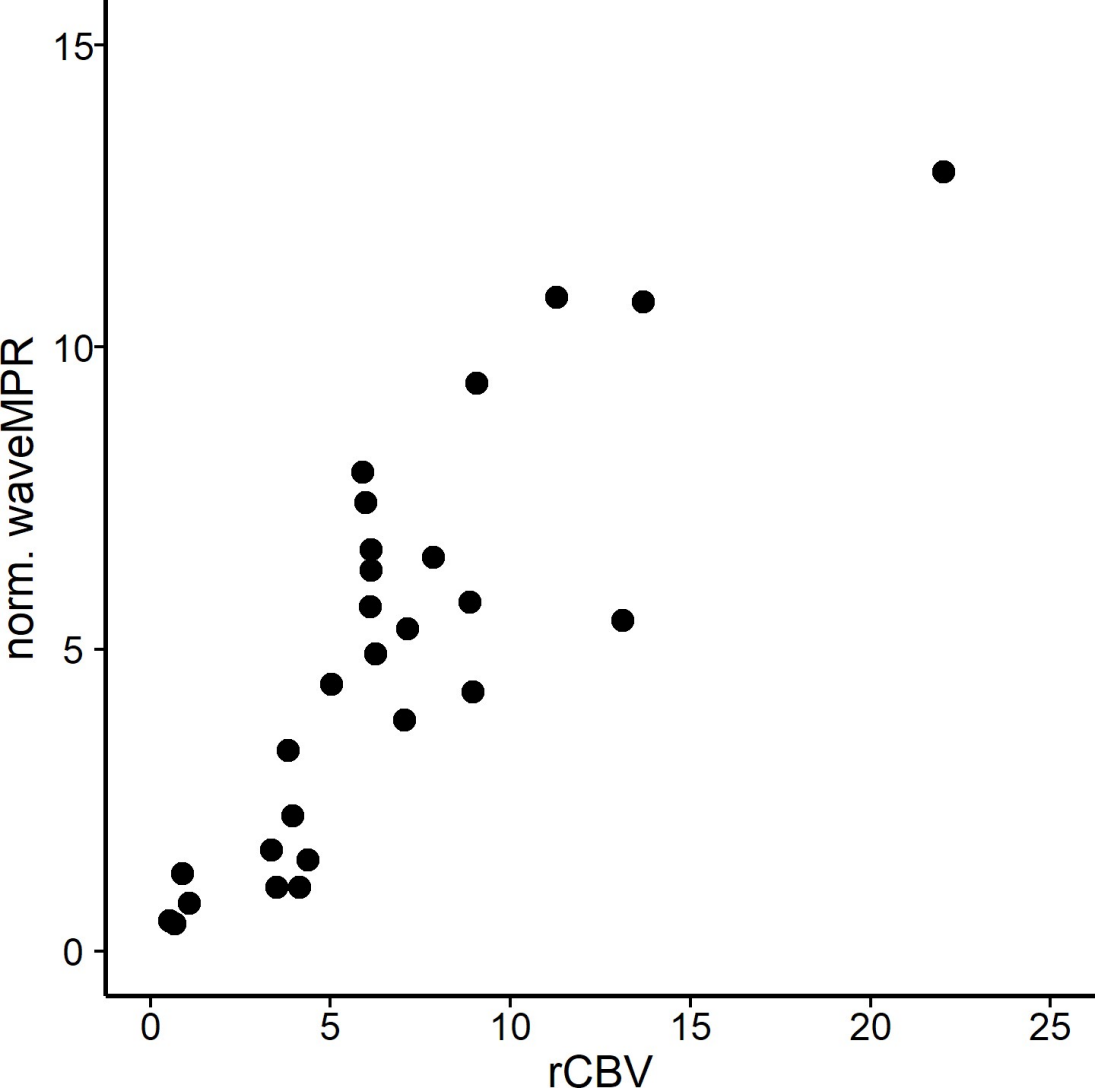
Scatterplot of normalized CBV versus normalized wavelet-MRP over all included biopsies.

In order to investigate the possible interchangeability of different perfusion parameters, we determined the linear association between wavelet-MRP and the established parameter rCBV. Pearson’s correlation coefficient was 0.81, indicating strong linear correlation. Testing this in a univariate linear model, we found the association to be highly significant (beta 1.13, p < 1e-7, Fig 4).

### Wavelet-MRP predicts local vessel density in a logistic regression model whereas rCBV does not

**Fig 5:**
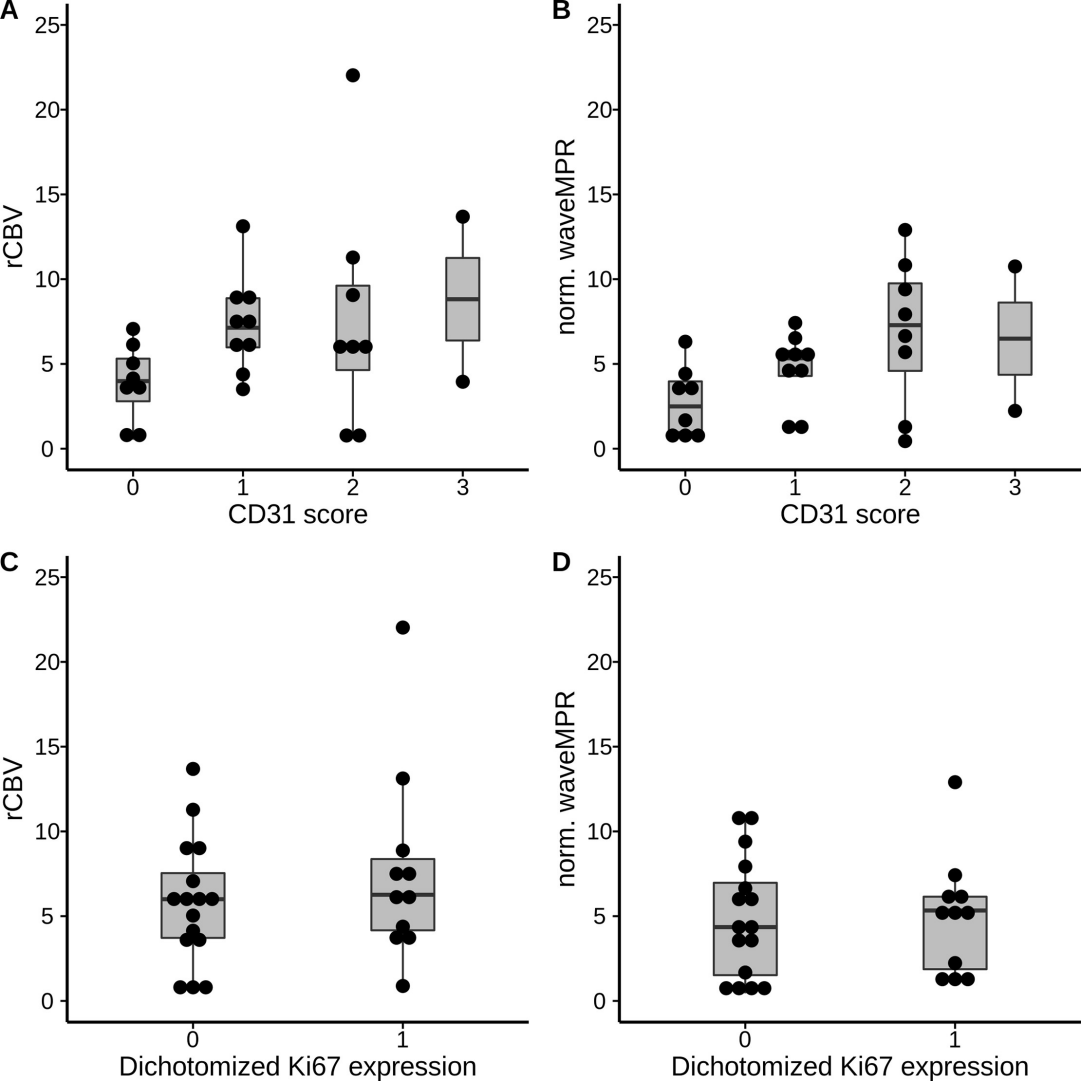
Box plots of normalized CBV (A) and normalized wavelet-MRP (B) in relation to the CD31 score as well as normalized CBV (C) and normalized wavelet-MRP (D) in relation to dichotomized Ki67-expression.

**Fig 6:**
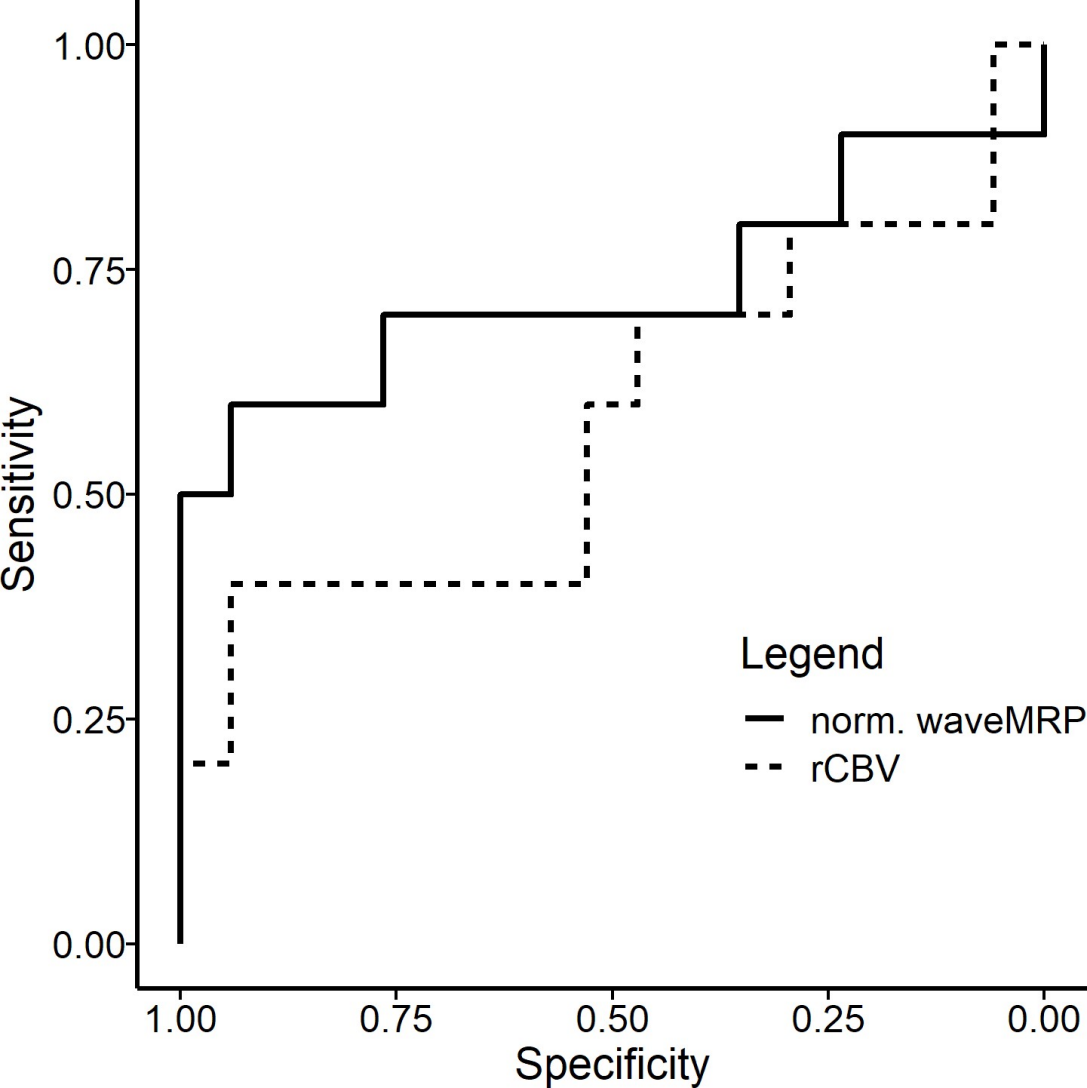
Receiver Operating Characteristic (ROC) curve for dichotomized CD31 expression and normalized CBV and normalized wavelet-MRP.

As described, local vessel density was assessed by visually grading the number of CD31-positive vessels on a four-point scale. 70% of the obtained samples showed nonzero expression. In a mixed logistic model with CD31 expression dichotomized to absent or present staining there was a significant association with wavelet-MRP (p = .043), whereas no significant association to rCBV could be found (p = .297). A description of the mixed logistic model can be found in Table 2. The Akaike Information Coeffiecient (AIC) was lower for wavelet-MRP (36.3) than for rCBV (39.7). Fig 5A and 5B show boxplots of local perfusion parameters dependent on sample CD31 and Fig 5C and 5D on sample Ki67 expression, respectively. In addition, Fig 6 shows a Reciever Operating Characteristic (ROC) curve for dichotomized CD31 expression and normalized CBV and normalized wavelet-MRP in a univariate model without random effects.

**Table 2:** Predictors of CD31 positivity. Mixed logistic regression with CD31 dichotomized to no or present staining as outcome and either rCBV or waveletMRP as predictor for all included matching tissue samples.

Independent variables	CE	p value	CE	p value
rCBV	0.11	0.297		
waveMRP			0.30	**0.043**
SE of Random Effect		0.19		0.73
AIC		39.7		36.3

CE coefficient estimate, rCBV relative Cerebral Blood Volume, wavelet-MRP wavelet-transformed Magnetic Resonance Perfusion, SE standard error, AIC Akaike Information Coefficient

### Tumor proliferation does not correlate with local perfusion magnitude

The mean proliferation index was 14% (14%), with 3 of the samples showing no proliferating cells. Neither in linear nor in nonlinear models a significant connection between Ki67 and rCBV (p = .62) or wavelet-MRP (p = .70) was found (Table 3).

**Table 3:** Predictors of MIB-1 staining ratio. Mixed linear regression with the Ki67 staining ratio as outcome and either rCBV or waveletMRP as predictor for all included matching tissue samples.

Independent variables	b	p value	b	p value
rCBV	0.003	0.62		
wavelet-MRP			0.003	0.70
SE of Random Effect		0.07		0.07
R^2^ (marginal)		0.01		0.006

rCBV relative Cerebral Blood Volume, wavelet-MRP wavelet-transformed Magnetic Resonance Perfusion, SE standard error

### Neither maximum nor mean proliferation correlate with perfusion indices

Additionally, we analyzed the potential relation of perfusion indices measured over the whole gross tumor volume to Ki67 and each other. Similar to the association seen when analyzing single VOIs, rCBV and wavelet-MRP showed high correlation (Pearsons correlation coefficient r = 0.73). Both parameters failed to show a significant association in a linear model with either maximal or mean Ki67 over GTV (p = .992 for rCBV, p = .899 for wavelet-MRP). Results can be found in S1 Table.

## Discussion

Microvascular proliferation (MVP) is a hallmark of GBM and an important parameter in the histopathological analysis of tumor tissue [14–16]. This study demonstrates that wavelet reconstructions of DSC PWI are associated with the number of CD31 positive vessels, and that wavelet-MRP might therefore serve as a surrogate for MVP. Advanced imaging techniques like PWI allow a noninvasive visualization of MVP as glioblastoma exhibits areas with high CBV values [17]. Wavelet-MRP is an innovative and alternative way of calculating vessel-specific color coded maps within from existing PWI data without the need for defining an arterial input function [7]. Wavelet-MRP can yield complementary information to CBV and shows higher image contrast and thus a higher contrast-to-noise ratio[7]. Therefore wavelet-MRP might be a beneficial additional tool in the visualization of MVP. The unique aspect of this study using a novel wavelet-transformed reconstruction in perfusion-weighted MRI [7] is the correlation with region-specific targeted biopsies that were obtained during neurosurgical tumor resections. Having human brain biopsy samples of clearly defined regions of interest from tumor and non-tumor regions in preoperative imaging is very rare. Therefore, existing data from the presented study cohort offered an almost ideal chance to substantiate our hypothesis that wavelet-MRP reflects vessel density. It has previously been shown that the wavelet post-processing technique can classify arterial and venous cerebral vessels in CT perfusion data with high statistical accuracy based on the time-domain wavelet transform of dynamic perfusion data [18]. Neoangiogenesis and MVP can lead to malfunctioning vessels in GBM with an abnormal flow-pattern. LaViolette et al. hypothesized that abnormal tumor vasculature with a higher ‘arterio-venous overlap’ (AVOL) exists in greater proportions in GB than in normal brain parenchyma and suggested AVOL as a potential parameter for therapy response. It is possible that wavelet-MRP could identify abnormal tumor-vessels based on the time-domain wavelet transform in PWI MRI. This would allow an even more tumor specific vascular visualization. The lower time resolutions of clinical MRI perfusion techniques compared to CT perfusion may pose technical constraints. As studies performed on CT perfusion datasets seem to indicate that lower time resolution does not have a clinically significant impact on perfusion parameters [19,20], it remains to be seen if this holds true for MR perfusion. Wavelet-MRP might further be valuable in the assessment of therapy response to anti–vascular endothelial growth factor (VEGF) antibodies like bevacizumab [3,21,22]. The low patient number is one of the major study limitations. However, study cohorts that included region-specific biopsies during neurosurgical resections are very rare. We used the number of stained vessels as a surrogate of microvasculature volume. It has been shown that other parameters, such as microvessel area, may provide better correlation with rCBV[23,24]. Still, this is expected to only lead to lower correlations than possible with other parameters. Another limitation of the study is the presence of brain shift during the biopsies which led to spatial imprecision. In contrast to stereotactic external biopsies, the samples in this study were taken during open brain surgery. After opening the skull brain shift could not be avoided and introduced imprecision between the planned region and the final region of biopsy. Exclusion of samples with apparent non-concordance was done to reduce bias due to spatial imprecision but can on the other hand introduce selection bias. Further, multiple biological samples were taken from the same individuals and these samples are non-independent. Though, for showing a correlation between perfusion parameters and histological findings we believe that this aspect is of inferior importance to the results of the present study. In addition to the use of wavelet-MRP in diagnostic, preoperative imaging, it could be an additional tool in the noninvasive postoperative tumor monitoring, for example to distinguish between radiation necrosis, pseudoprogression and early recurrent tumor, since the latter is correlates with a very poor prognosis and requires a fast change of therapy [25]. Advanced imaging techniques like MR spectroscopy, diffusion weighted imaging and CBV measurements obtained by DSC are often applied for this purpose [17,26–31]. For instance, higher CBV values are typically associated with a higher chance of tumor recurrence, due to the higher amount of MVP [17]. The results of our study suggest that wavelet-MRP can serve as a new and beneficial surrogate for MVP in GBM as well. In contrast to CBV, the wavelet-MRP is a unit-free parameter and should be calculated in relation to a physiological brain structure like the normal white matter as it was done in our study. Wavelet-MRP might also be valuable in the planning of stereotactic biopsies since it can illustrate MVP hot-spots and hemodynamic tissue information exhibit prognostic capabilities in GBM [32]. The ROC curve for dichotomized CD31 expression revealed a benefit of wavelet-MRP over rCBV mainly in regions of high specificity, indicating that wavelet-MRP may be able to find spots of abnormal perfusion that would be otherwise missed. This could stem contrast extravasation algorithms interfering with detection of small regions. While leakage-correction methods were applied during postprocessing, perfusion measurements can suffer inaccuracies when no pre-bolus is applied [33]. Further studies on wavelet-MRP in malignant brain processes are necessary to further elucidate the value of this alternative post-processing technique.

## Conclusions

Wavelet-MRP can be calculated from existing MR perfusion data and might be a new surrogate for tumor vascularity in GBM as the wavelet-MRP power spectrum correlates with the endothelial marker CD31.

## Supporting information

S1 Table Univariate linear regression with the Ki67 staining ratio as outcome and either mean rCBV or waveletMRP over GTV as predictor. (DOCX)

## References

[1] Leu K, Enzmann DR, Woodworth DC, Harris RJ, Tran AN, Lai A, et al Hypervascular tumor volume estimated by comparison to a large-scale cerebral blood volume radiographic atlas predicts survival in recurrent glioblastoma treated with bevacizumab. Cancer Imaging. 2014;14: 31 10.1186/s40644-014-0031-z 25608485 PMC4331836

[2] Jain R, Griffith B, Alotaibi F, Zagzag D, Fine H, Golfinos J, et al Glioma angiogenesis and perfusion imaging: Understanding the relationship between tumor blood volume and leakiness with increasing glioma grade. Am J Neuroradiol. 2015;36: 2030–2035. 10.3174/ajnr.A4405 26206809 PMC7964882

[3] Singh R, Kesavabhotla K, Kishore SA, Zhou Z, Tsiouris AJ, Filippi CG, et al Dynamic susceptibility contrast-enhanced mr perfusion imaging in assessing recurrent glioblastoma response to superselective intra-arterial bevacizumab therapy. Am J Neuroradiol. 2016;37: 1838–1843. 10.3174/ajnr.A4823 27231225 PMC5124417

[4] Bennett IE, Field KM, Hovens CM, Moffat BA, Rosenthal MA, Drummond K, et al Early perfusion MRI predicts survival outcome in patients with recurrent glioblastoma treated with bevacizumab and carboplatin. J Neurooncol. 2017;131: 321–329. 10.1007/s11060-016-2300-0 27896520

[5] Havla L, Thierfelder KM, Beyer SE, Sommer WH, Dietrich O. Wavelet-based calculation of cerebral angiographic data from time-resolved CT perfusion acquisitions. Eur Radiol. 2015;25: 2354–2361. 10.1007/s00330-015-3651-1 25716940

[6] Kunz WG, Sommer WH, Havla L, Dorn F, Meinel FG, Dietrich O, et al Detection of single-phase CTA occult vessel occlusions in acute ischemic stroke using CT perfusion-based wavelet-transformed angiography. Eur Radiol. 2017;27: 2657–2664. 10.1007/s00330-016-4613-y 27722798

[7] Huber T, Rotkopf L, Wiestler B, Kunz WG, Bette S, Gempt J, et al Wavelet-based reconstruction of dynamic susceptibility MR-perfusion: a new method to visualize hypervascular brain tumors. Eur Radiol. 2019;29: 2669–2676. 10.1007/s00330-018-5892-2 30552476

[8] Preibisch C, Shi K, Kluge A, Lukas M, Wiestler B, Göttler J, et al Characterizing hypoxia in human glioma: A simultaneous multimodal MRI and PET study. NMR Biomed. 2017;30: e3775 10.1002/nbm.3775 28805936

[9] Kluge A, Lukas M, Toth V, Pyka T, Zimmer C, Preibisch C. Analysis of three leakage-correction methods for DSC-based measurement of relative cerebral blood volume with respect to heterogeneity in human gliomas. Magn Reson Imaging. 2016;34: 410–421. 10.1016/j.mri.2015.12.015 26708034

[10] Hedderich D, Kluge A, Pyka T, Zimmer C, Kirschke JS, Wiestler B, et al Consistency of normalized cerebral blood volume values in glioblastoma using different leakage correction algorithms on dynamic susceptibility contrast magnetic resonance imaging data without and with preload. J Neuroradiol. 2019;46: 44–51. 10.1016/j.neurad.2018.04.006 29753641

[11] Coşkun E, Özder S, Tiryaki E. The Paul wavelet algorithm: An alternative approach to calculate the refractive index dispersion of a dielectric film from transmittance spectrum. Appl Phys B Lasers Opt. 2013;113: 243–250. 10.1007/s00340-013-5465-7

[12] Torrence C, Compo GP. A Practical Guide to Wavelet Analysis. Bull Am Meteorol Soc. 1998;79: 61–78. 10.1175/1520-0477(1998)079<0061:APGTWA>2.0.CO;2

[13] Woodworth G, McGirt MJ, Samdani A, Garonzik I, Olivi A, Weingart JD. Accuracy of frameless and frame-based image-guided stereotactic brain biopsy in the diagnosis of glioma: Comparison of biopsy and open resection specimen. Neurol Res. 2005;27: 358–362. 10.1179/016164105X40057 15949232

[14] Louis DN, Ohgaki H, Wiestler OD, Cavenee WK, Burger PC, Jouvet A, et al The 2007 WHO classification of tumours of the central nervous system. Acta Neuropathol. 2007;114: 97–109. 10.1007/s00401-007-0243-4 17618441 PMC1929165

[15] Brat DJ, Aldape K, Colman H, Holland EC, Louis DN, Jenkins RB, et al cIMPACT-NOW update 3: recommended diagnostic criteria for “Diffuse astrocytic glioma, IDH-wildtype, with molecular features of glioblastoma, WHO grade IV.” Acta Neuropathol. 2018;136: 805–810. 10.1007/s00401-018-1913-0 30259105 PMC6204285

[16] Louis DN, Perry A, Reifenberger G, von Deimling A, Figarella-Branger D, Cavenee WK, et al The 2016 World Health Organization Classification of Tumors of the Central Nervous System: a summary. Acta Neuropathol. 2016;131: 803–820. 10.1007/s00401-016-1545-1 27157931

[17] Hu LS, Baxter LC, Smith KA, Feuerstein BG, Karis JP, Eschbacher JM, et al Relative cerebral blood volume values to differentiate high-grade glioma recurrence from posttreatment radiation effect: Direct correlation between image-guided tissue histopathology and localized dynamic susceptibility-weighted contrast-enhanced perfusio. Am J Neuroradiol. 2009;30: 552–558. 10.3174/ajnr.A1377 19056837 PMC7051449

[18] Havla L, Schneider MJ, Thierfelder KM, Beyer SE, Ertl-Wagner B, Reiser MF, et al Classification of arterial and venous cerebral vasculature based on wavelet postprocessing of CT perfusion data. Med Phys. 2016;43: 702–709. 10.1118/1.4939224 26843234

[19] Wintermark M, Smith WS, Ko NU, Quist M, Schnyder P, Dillon WP. Dynamic perfusion CT: Optimizing the temporal resolution and contrast volume for calculation of perfusion CT parameters in stroke patients. Am J Neuroradiol. 2004;25: 720–729. 15140710 PMC7974477

[20] Abels B, Klotz E, Tomandl BF, Villablanca JP, Kloska SP, Lell MM. CT perfusion in acute ischemic stroke: A comparison of 2-second and 1-second temporal resolution. Am J Neuroradiol. 2011;32: 1632–1639. 10.3174/ajnr.A2576 21816919 PMC7965399

[21] Ameratunga M, Pavlakis N, Wheeler H, Grant R, Simes J, Khasraw M. Anti-angiogenic therapy for high-grade glioma. Cochrane Database of Systematic Reviews. 2018 10.1002/14651858.CD008218.pub4 30480778 PMC6516839

[22] Ken S, Deviers A, Filleron T, Catalaa I, Lotterie JA, Khalifa J, et al Voxel-based evidence of perfusion normalization in glioblastoma patients included in a phase I–II trial of radiotherapy/tipifarnib combination. J Neurooncol. 2015;124: 465–473. 10.1007/s11060-015-1860-8 26189058

[23] Hu LS, Eschbacher JM, Dueck AC, Heiserman JE, Liu S, Karis JP, et al Correlations between perfusion MR imaging cerebral blood volume, microvessel quantification, and clinical outcome using stereotactic analysis in recurrent high-grade glioma. Am J Neuroradiol. 2012;33: 69–76. 10.3174/ajnr.A2743 22095961 PMC7966183

[24] Pathak AP, Schmainda KM, Douglas Ward B, Linderman JR, Rebro KJ, Greene AS. MR-derived cerebral blood volume maps: Issues regarding histological validation and assessment of tumor angiogenesis. Magn Reson Med. 2001;46: 735–747. 10.1002/mrm.1252 11590650

[25] Melguizo-Gavilanes I, Bruner JM, Guha-Thakurta N, Hess KR, Puduvalli VK. Characterization of pseudoprogression in patients with glioblastoma: is histology the gold standard? J Neurooncol. 2015;123: 141–150. 10.1007/s11060-015-1774-5 25894594 PMC4780341

[26] Zeng QS, Li CF, Liu H, Zhen JH, Feng DC. Distinction Between Recurrent Glioma and Radiation Injury Using Magnetic Resonance Spectroscopy in Combination With Diffusion-Weighted Imaging. Int J Radiat Oncol Biol Phys. 2007;68: 151–158. 10.1016/j.ijrobp.2006.12.001 17289287

[27] Zeng QS, Li CF, Zhang K, Liu H, Kang XS, Zhen JH. Multivoxel 3D proton MR spectroscopy in the distinction of recurrent glioma from radiation injury. J Neurooncol. 2007;84: 63–69. 10.1007/s11060-007-9341-3 17619225

[28] Bette S, Huber T, Gempt J, Boeckh-Behrens T, Wiestler B, Kehl V, et al Local fractional anisotropy is reduced in areas with tumor recurrence in glioblastoma. Radiology. 2017;283: 499–507. 10.1148/radiol.2016152832 28234549

[29] Hojjati M, Badve C, Garg V, Tatsuoka C, Rogers L, Sloan A, et al Role of FDG-PET/MRI, FDG-PET/CT, and Dynamic Susceptibility Contrast Perfusion MRI in Differentiating Radiation Necrosis from Tumor Recurrence in Glioblastomas. J Neuroimaging. 2018;28: 118–125. 10.1111/jon.12460 28718993 PMC5811794

[30] Chuang MT, Liu YS, Tsai YS, Chen YC, Wang CK. Differentiating radiation-induced necrosis from recurrent brain tumor using MR perfusion and spectroscopy: A meta-analysis. Hendrikse J, editor. PLoS One. 2016;11: e0141438 10.1371/journal.pone.0141438 26741961 PMC4712150

[31] Young RJ, Gupta A, Shah AD, Graber JJ, Chan TA, Zhang Z, et al MRI perfusion in determining pseudoprogression in patients with glioblastoma. Clin Imaging. 2013;37: 41–49. 10.1016/j.clinimag.2012.02.016 23151413 PMC4755513

[32] Juan-Albarracín J, Fuster-Garcia E, Pérez-Girbés A, Aparici-Robles F, Alberich-Bayarri Á, Revert-Ventura A, et al Glioblastoma: Vascular Habitats Detected at Preoperative Dynamic Susceptibility-weighted Contrast-enhanced Perfusion MR Imaging Predict Survival. Radiology. 2018;287: 944–954. 10.1148/radiol.2017170845 29357274

[33] Welker K, Boxerman J, Kalnin A, Kaufmann T, Shiroishi M, Wintermark M. ASFNR recommendations for clinical performance of MR dynamic susceptibility contrast perfusion imaging of the brain. Am J Neuroradiol. 2015;36: E41–E51. 10.3174/ajnr.A4341 25907520 PMC5074767

