# Supplementary material for: The wavelet power spectrum of perfusion weighted MRI correlates with tumor vascularity in biopsy-proven glioblastoma samples
Source: PLoS One. 2020 Jan 23;15(1):e0228030. doi: 10.1371/journal.pone.0228030 (PMC6977746; doi:10.1371/journal.pone.0228030)
Supplement: S1 Table — (DOCX) [file pone.0228030.s001.docx]

**Supplement 1: Predictors of mean Ki67 staining ratio**

|  |  |  |  |  |
| --- | --- | --- | --- | --- |
| Independent variables | b | p value | b | p value |
|  |  |  |  |  |
| rCBV | 0.000 | 0.992 | - |  |
| wavelet-MRP |  |  | 0.001 | 0.899 |
|  |  |  |  |  |
| R^2^ |  | <0.001 |  | 0.002 |
|  |  |  |  |  |
|  |  |  |  |  |

rCBV relative Cerebral Blood Volume, wavelet-MRP wavelet-transformed Magnetic Resonance Perfusion
